# Frontline treatment with chemoimmunotherapy for limited-stage ocular adnexal MALT lymphoma with adverse factors: a phase II study

**DOI:** 10.18632/oncotarget.19788

**Published:** 2017-08-02

**Authors:** Sung-Yong Kim, Suk-Woo Yang, Won-Sik Lee, Jae Wook Yang, Sung Yong Oh, Hee Bae Ahn, Deok-Hwan Yang, Seong Kyu Park, Jee Ho Chang, Hyo Jung Kim, Min Joung Lee, Seok-Goo Cho

**Affiliations:** ^1^ Department of Hematology-Oncology, Konkuk University Medical Center, Konkuk University School of Medicine, Seoul, Korea; ^2^ Department of Ophthalmology and Visual Science, Seoul St. Mary’s Hospital, The Catholic University of Korea College of Medicine, Seoul, Korea; ^3^ Department of Hematology and Oncology, Inje University College of Medicine, Busan Paik Hospital, Busan, Korea; ^4^ Department of Ophthalmology, Busan Paik Hospital, Inje University, Busan, Korea; ^5^ Department of Internal Medicine, Dong-A University College of Medicine, Busan, Korea; ^6^ Department of Ophthalmology, Dong-A University College of Medicine, Busan, Korea; ^7^ Department of Hematology-Oncology, Chonnam National University Hwasun Hospital, Jeollanamdo, Korea; ^8^ Department of Internal Medicine, Soonchunhyang University Bucheon Hospital, Soonchunhyang University, Bucheon, Korea; ^9^ Department of Ophthalmology, Soonchunhyang University Bucheon Hospital, Soonchunhyang University, Bucheon, Korea; ^10^ Division of Hematology-Oncology, Department of Internal Medicine, College of Medicine, Hallym University, Hallym University Sacred Heart Hospital, Anyang, Korea; ^11^ Department of Ophthalmology, College of Medicine, Hallym University, Hallym University Sacred Heart Hospital, Anyang, Korea; ^12^ Department of Hematology, Catholic Blood and Marrow Transplantation Center, Seoul St. Mary's Hospital, The Catholic University of Korea, Seoul, Korea

**Keywords:** lymphoma, ocular, mucosa associated lymphoid tissue, rituximab, chemoimmunotherapy

## Abstract

**Background:**

Radiotherapy is a commonly used treatment for limited-stage ocular adnexal mucosa-associated lymphoid tissue lymphoma (OAML) but showed a substantial relapse risk if the disease involves beyond-conjunctiva or bilateral conjunctivae. Systemic chemoimmunotherapy may be an alternative frontline therapy for the limited disease with those adverse prognostic factors.

**Patients and methods:**

We designed a multicenter, phase II study of the chemoimmunotherapy, rituximab, cyclophosphamide, vincristine, and prednisolone (R-CVP) for the treatment of patients with limited-stage OAML with bilateral or beyond-conjunctival involvement. Thirty-three patients with Ann Arbor stage I OAML with the adverse factors were enrolled. Patients received six cycles of R-CVP followed by two cycles of rituximab therapy.

**Results:**

At the end of treatment, all the enrolled patients had responded. The cumulative complete response achievement was 93.9% at 2 years. At a median follow-up of 50.6 months, three patients had progressed. Progression-free survival and overall survival at 4 years was 90.3±5.3% and 100%, respectively.

**Conclusions:**

This phase II study demonstrated durable efficacy of R-CVP chemoimmunotherapy, which has promise as an alternative frontline therapy for the limited-stage OAML patients with adverse prognostic factors.

**Clinical trial registration:**

NCT01427114.

## INTRODUCTION

Ocular adnexal mucosa-associated lymphoid tissue (MALT) lymphoma (OAML) is the most common type of ocular lymphoma and its prevalence is higher in Asia than in western countries [1–3]. OAML is a slowly growing disease and it is generally responsive to radiation therapy. Therefore, patients with limited-stage disease usually undergo radiation therapy, while systemic chemotherapy has been rarely tested as a frontline therapy. However, limited-stage OAML has been reported to have a recurrence rate of 25% following radiotherapy [4], and the risk of recurrence is higher when the disease involves both eyes or spreads beyond the conjunctiva [5–8]. Previous studies using radiotherapy have demonstrated that the disease recurred predominantly in areas that were not irradiated, mostly the contralateral eye and distant extranodal organs [7, 9–12], so that we reasoned that the relapse rate of nonirradiated areas might be reduced if the limited staged patients with the higher risk factors receive systemic chemoimmunotherapy rather than radiotherapy. In addition, the target volume of irradiation for conjunctiva-only disease is the whole conjunctiva and the risk of cataract can be reduced with lens shielding, but for disease extending beyond the conjunctiva, the whole orbit is generally covered without lens shielding so that the risk of cataract can be considerable [9, 13, 14].

However, only a few trials of systemic chemotherapy or chemoimmunotherapy as a frontline treatment have been conducted in OAML patients with limited-stage disease, because of the rarity of the disease and its responsiveness to radiation therapy [15–17]. Furthermore, most of the prior studies using systemic chemotherapy or chemoimmunotherapy included advanced stage OAML or MALT lymphoma of other sites, and thus it is hard to estimate the efficacy of the frontline systemic therapy in limited-stage OAML. Before rituximab was introduced, a prior retrospective study demonstrated the efficacy of combination chemotherapy, cyclophosphamide, vincristine, and prednisolone (CVP) for the frontline treatment of limited staged OAML [18]. This study is the only previous study of patients with limited-stage OAML who were treated with systemic therapy as a frontline treatment; the complete response (CR) rate for limited-stage OAML with beyond-conjunctival involvement was 67% and the progression-free survival (PFS) was disappointing. Recently, rituximab has been used for treatment of OAML, but rituximab monotherapy did not demonstrate adequate efficacy, with frequent early relapse and often distant relapse [17]. These observations suggested the need for a combination of rituximab and conventional cytotoxic chemotherapy to ensure long-term disease control. Several prospective trials using rituximab-containing chemoimmunotherapy were conducted for extranodal marginal zone B cell lymphoma, including the MALT-2008-01 and IELSG-19 trials but these studies enrolled various stage and site MALT lymphoma patients [15, 16, 19, 20].

In this phase II study, a patient with newly diagnosed limited-stage OAML involving bilateral ocular adnexae or spreading beyond the conjunctiva was treated with a combination chemoimmunotherapy with rituximab and CVP, which is generally used in advanced stage, slowly growing CD20 + B cell lymphoma [21]. The present study was designed based on a previous study testing the CVP regimen for limited-stage OAML, and aimed to evaluate the efficacy of this regimen as a frontline therapy for the treatment of limited-stage OAML with adverse factors. This trial was registered with the National Cancer Institute (http://www.clinicaltrials.gov; identifier NCT01427114).

## RESULTS

### Patients and disease

The study patients and disease characteristics are described in Table 1. A total of 33 patients, 21 men and 12 women, were enrolled in this study and all completed the planned treatment. The age of the study patients was 49 years (range, 19–74 years). All study patients had good performance scores and did not have B symptoms. Three patients had elevated levels of lactate dehydrogenase (LDH) while all other study patients had normal levels of LDH.

**Table 1:** Study patients and disease characteristics

Characteristics	Number (%)
Total	33
Gender	
Male	21 (64%)
Female	12 (36%)
Age, median (range)	49 years (19-74)
Lactic dehydrogenase	
Elevated	3 (9%)
Normal	30 (91%)
B symptom	
Yes	0 (0%)
No	33 (100%)
ECOG Performance Status	
0-1	33 (100%)
2-4	0 (0%)
Location	
Orbit	13 (39.4%)
Conjunctiva	12 (36.4%)
Eyelid	5 (15.2%)
Lacrimal gland or duct	3 (9.1%)
Ann Arbor stage	
IE	33 (100%)
IIE	0 (0%)
TNM stage*	
T1N0M0	0 (0%)
bT1N0M0	10 (30.3%)
T2N0M0	16 (48.5%)
bT2N0M0	3 (9.1%)
T3N0M0	2 (6.1%)
bT3N0M0	1 (3%)
T4N0M0	1 (3%)
bT4N0M0	0 (0%)

* TNM stage was based on the clinical staging system of ocular adnexal lymphoma proposed by the American Joint Committee on Cancer. *ECOG; Eastern Cooperative Oncology Group*.

The anatomic location of disease was the conjunctiva in 12 patients (36.4%), the orbit in 13 (39.4%), the eyelid in five (15.2%), and the lacrimal duct or gland in three (9.1%). Fourteen patients (42.4%) had bilateral disease at presentation. According to the Ann Arbor staging at the time of diagnosis, all patients enrolled had stage IE disease, although the eligibility included stage I and II disease. Based on the tumor–node–metastasis (TNM) staging [22], 10 (30.3%) patients had T1 disease with bilateral involvement and the remaining 23 patients (69.7%) had T2 or higher disease.

### Response and cumulative incidence of CR achievement

All study patients responded to the treatment (Table 2). After the third cycle of R-CVP, 15 patients (45.5%) achieved CR and 18 (54.5%) partial response (PR). At one month after the treatment, 28 patients (84.8%) were in CR and five patients (15.2%) in PR. On follow up, 31 patients (93.9%) had achieved CR at best and two (6.1%) remained in PR. The median time to CR was 4.1 months (range, 1.7–17.7 months) after initiation of treatment in patients who achieved CR. Because the study population was small and the CR rate was high, no significant predictors of best response were identified by statistical analysis.

**Table 2:** Response to R-CVP in limited stage ocular adnexal MALT lymphoma patients with bilateral or extra-conjunctival involvement

	1 month after the completion of treatment	Best response during follow up	Last follow up
CR	28 (84.8%)	31 (93.9%)	30 (90.9%)
PR	5 (15.2%)	2 (6.1%)	2 (6.1%)
NR	0 (0%)	0 (0%)	0 (0%)
Progression	0 (0%)	0 (0%)	3 (9.1%)*

* Three patients relapsed 22.5, 35.1 and 37.4 months of treatment, respectively. *CR: complete response; PR: partial response; NR: no response; LDH: lactic dehydrogenase; IPI: international prognostic index*.

**Figure 1:**
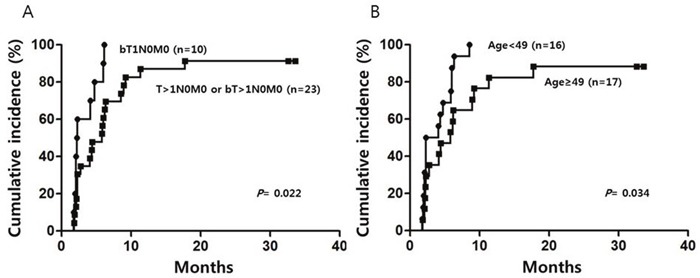
Cumulative incidence of complete response in the study patients according to TNM group **(A)** and age group **(B)**, assessed by univariate analysis.

The median duration of follow-up was 50.6 months (range, 7.4-62.8 months). The cumulative CR rate was 93.9% at 2 years. The cumulative CR rate in patients who had conjunctiva-confined disease (T=1) was significantly higher than that in patients who had disease spreading beyond the conjunctiva (T>1) (100% vs. 91.3%; P = 0.022, Figure 1). Patients under 49 years of age had higher CR rates than patients over 49 years of age (100% vs. 90.4%; P = 0.034). The involvement of areas beyond the conjunctiva (T>1) was the only independent factor for CR acquisition by the multivariate analysis (hazard ratio [HR] 0.424, P = 0.044).

### Progression-free and overall survival

**Figure 2:**
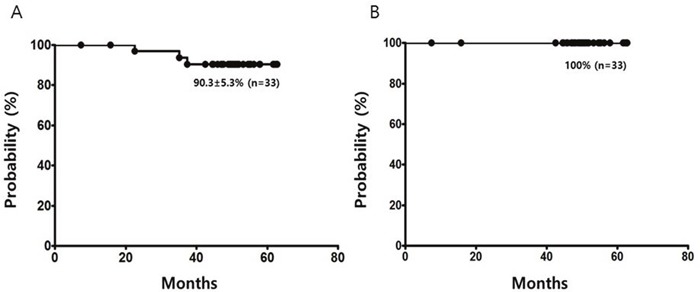
Progression-free survival **(A)** and overall survival **(B)** of limited stage ocular adnexal MALT lymphoma patients with bilateral or beyond-conjunctival involvement treated with R-CVP.

Among the study patients, three patients relapsed at 22.5, 35.1, and 37.4 month after initiation of treatment whose TNM stage was bT1, T2a, and T2d, respectively. All the three patients relapsed at the same location in the eye and thus received localized radiotherapy as a second line treatment. They were currently in CR at last follow-up. No study patient showed distant relapse during follow up. The estimated PFS at 4 years was 90.3 ± 5.3% (Figure 2). None of the study patients died until the last observation, and overall survival (OS) was 100%. No significant prognostic factors for OS and PFS were identified by the statistical analysis.

### Toxicity

The treatment-related toxicities are listed in Table 3. All study patients completed the treatment following the planned doses without dose reduction. The common frequent side effects of this regimen were neutropenia and peripheral neuropathy. Four patients experienced grade 3 or 4 neutropenia and two patients had grade 3 or 4 hepatotoxicity. No patient underwent cataract surgery and had secondary malignancy until last day of follow-up.

**Table 3:** Toxicity

Toxicity	Any grade	Grade 3 or 4
Anemia	6 (19%)	0 (0%)
Neutropenia	11 (33%)	4 (12%)
Paresthesia	11 (33%)	0 (0%)
Hepatotoxicity	7 (21%)	2 (6%)
Constipation	4 (12%)	0 (0%)
Hyperglycemia	3 (9%)	1 (3%)

## DISCUSSION

Previous series showed that 7-22% of OAML patients have bilaterality and 29-73% of OAML patients have non-conjunctival disease [8]. We thought that these patients would benefit most from this phase II study because this group of patients has a higher risk of relapse and radiation related complications after radiotherapy. The overall response rate to R-CVP treatment for limited-stage OAML with these adverse factors was 100%, with CR of 84.8%. The overall CR rate reached 93.9% at 2 years and the PFS was 90.3% at 4 years. Of note, all relapse sites were at the original locations and distant relapse was not observed in any of our study patients. Previous studies using radiotherapy have demonstrated that the disease recurred predominantly in non-irradiated contralateral and distant areas but we did not observe distant recurrence in our study patients, which suggests that systemic chemoimmunotherapy may reduce the risk of distant recurrence.

Because prior trials using either rituximab or cytotoxic agents alone for MALT lymphoma had not shown satisfactory results [17, 18, 23], recent studies have evaluated combination regimens consisting of rituximab plus cytotoxic chemotherapeutic agents for the treatment of patients with various sites and stages I-IV of MALT lymphoma and shown promising results [15, 16, 20]. A previous phase II study, MALT2008-01, using rituximab plus bendamustine in patients with MALT lymphoma reported a CR rate of 100% and a 4-year PFS of 91% in non-gastric disease. Another previous phase III study, IELSG-19, using rituximab plus chlorambucil for extranodal marginal zone B cell lymphoma reported a CR rate of 78% and 5-year PFS of 71% [15, 16]. Although direct comparison with other chemoimmunotherapy including rituximab is difficult because of heterogeneity of the prior study patients including gastric MALT lymphoma and stage III-IV disease patients, the present trial suggested R-CVP regimen has comparable treatment outcomes to those for other chemoimmunotherapy regimens. However, many previous reports suggested that MALT lymphoma might progress a long time after achieving CR or PR with radiotherapy or systemic therapy [1, 11, 12, 16] and thus we need further follow up to confirm an excellent long-term PFS in this group of patients when they are treated with R-CVP or other chemoimmunotherapy.

The overall response rate and OS in this study was 100% and three patients relapsed; hence, predictors of response, OS, and PFS were not identifiable by statistical analysis but a predictor for cumulative CR achievement was identified. The patients with disease involving structures beyond conjunctivae (T>1) showed a lower cumulative CR rate, even after adjusting for other potential prognostic factors. Ocular adnexal lymphoma is generally staged using the Ann Arbor staging system as other lymphoma, but this system is not perfect for this disease because most patients with this disease have limited stage and the bilaterality, nonconjunctival location, and nodal involvement are related to a worse prognosis in limited-stage patients [8]. Our previous study also suggested that the treatment outcome of OAML can be influenced by the TNM stage when the disease is treated by radiotherapy [6]. The present study also suggests that the TNM stage could have an independent impact on CR achievement in patients with limited-stage disease according to the Ann Arbor staging system, even when they are treated with systemic chemoimmunotherapy. Older age was likely to be another unfavorable predictor for CR achievement, with borderline significance (*P* = 0.062). Because we could not identify any prognostic factors for PFS, including TNM stage, further follow up is required to clarify whether T>1 by the TNM staging system can be associated with lower PFS.

The R-CVP regimen is a widely used treatment for lymphoma and the toxicities in the present study were as observed in previous studies [24]. We did not observe any ophthalmic complications, which demonstrated that R-CVP can avoid the radiotherapy-related ophthalmic complications. This finding was consistent with the previous retrospective studies, in which patients treated with chemotherapy alone showed few of the ophthalmic complications that were frequently seen after radiotherapy [18, 23, 25].

This phase II study demonstrated that R-CVP is a more-effective frontline regimen showing durable response for limited-stage OAML with adverse factors than is CVP. The chemoimmunotherapy can be considered as the alternative frontline therapy for limited-stage OAML patients who wish to avoid the risk of radiologic ophthalmic complications.

## MATERIALS AND METHODS

### Patients and study design

This single arm, open-label, multicenter, phase II clinical trial evaluated R-CVP treatment in limited-stage OAML with bilateral or beyond-conjunctival involvement. Patients with a histologically confirmed extranodal marginal zone B-cell lymphoma of MALT from the ocular adnexa were included.

The enrolment criteria for disease status were Ann Arbor stage I–II OAML with adverse factors; bilateral or beyond-conjunctival involvement, which is bT1 or T>1, N0, and M0 based on the TNM staging system for ocular adnexal lymphoma proposed by the American Joint Committee on Cancer. Measurable disease at baseline was defined as at least one lesion that was accurately measurable. Patients were required to be ≥ 18 years old, with Eastern Cooperative Oncology Group (ECOG) performance scores of 0–2, with no prior chemotherapy or radiation therapy, and with adequate bone marrow, renal and hepatic function. Exclusions were made for disease confined to unilateral conjunctiva (T1N0M0) or Ann Arbor stage III–IV disease. In this study, bilateral disease without involvement of any other organ was considered as stage IE rather than stage IVE.

The protocol was approved by the Institutional Review Boards of all participating institutions and all patients provided written informed consent at the time of enrolment. We described the study to consecutive patients who were eligible for this study, and if the patients agreed to participate in this study, we enrolled them.

### Treatment

Patients were treated every 21 days with six cycles of rituximab (375 mg/m^2^), cyclophosphamide (750 mg/m^2^), and vincristine (1.4 mg/m^2^) on day 1 and prednisolone (60 mg/m^2^) on days 1–5, which was followed by 2 cycles of rituximab (375 mg/m^2^) every 21 days. Dose adjustment was permitted based on hematologic toxicity, neurologic toxicity, and infusion reactions. For hematologic toxicity, the use of granulocyte colony-stimulating factor was allowed.

### Study end points and assessments

The primary end point of the study was the CR rate based on the revised response criteria for malignant lymphoma [26]. The secondary end point was PFS and OS.

For the evaluation of efficacy, a contrast-enhanced magnetic resonance imaging (MRI) scan and ophthalmic examination were performed at baseline, within four weeks of the start of treatment. Subsequently, the same imaging study and ophthalmic exam were repeated for response assessment after the third and sixth cycles of R-CVP and after completion of all planned treatments. Follow-up reassessments were conducted every 3 months during the first 2 years and every 6 months thereafter. We did not recommend routine follow-up bone marrow study for this limited-stage disease except at the initial baseline work up. Follow-up bone marrow biopsy was planned only when the disease progressed or relapsed. Treatment-related toxicities were evaluated using the Common Terminology Criteria for Adverse Events (CTCAE 4.0).

### Statistical considerations

A single-stage design was used to evaluate the efficacy of the regimen. A CR rate of ≥ 87% among patients at the end of the study was considered superior to that for the CVP regimen. The target rates were based on the study by Song et al. which showed a 67% CR rate to CVP in patients with limited-stage bilateral or beyond-conjunctival OAML [18]. We thus needed 29 evaluable patients with a one-sided type I error of 5% and 80% power for a single-stage design [27, 28]. Anticipating a 10% drop-out rate, we enrolled 33 patients.

PFS was defined as the time from entry onto this study until lymphoma progression or death as a result of any cause. OS was defined as the time from entry onto this study until death as a result of any cause and censored at the date of the last follow-up visit [26]. Time to CR was defined as the time from the date of treatment initiation to the date of documented CR. The cumulative incidence of CR was calculated using time to CR. Cox regression hazard analysis was used for multivariate analysis of survival outcomes and the cumulative incidence of CR achievement. A 2-sided *P* value < 0.05 was considered significant.
